# What Took Them So Long? Explaining PhD Delays among Doctoral Candidates

**DOI:** 10.1371/journal.pone.0068839

**Published:** 2013-07-23

**Authors:** Rens van de Schoot, Mara A. Yerkes, Jolien M. Mouw, Hans Sonneveld

**Affiliations:** 1 Department of Methods and Statistics, Utrecht University, Utrecht, The Netherlands; 2 Optentia Research Focus Area, North-West University, Vanderbijlpark, South Africa; 3 Institute for Social Science Research, University of Queensland, Brisbane, Australia; 4 Erasmus University Rotterdam, Rotterdam, The Netherlands; 5 Education and Child Studies, Faculty of Social and Behavioural Sciences, Leiden University, Leiden, The Netherlands; 6 Netherlands Centre for Graduate and Research Schools, Utrecht, The Netherlands; 7 Tilburg Law School, Tilburg University, Tilburg, The Netherlands; University of Florida, United States of America

## Abstract

A delay in PhD completion, while likely undesirable for PhD candidates, can also be detrimental to universities if and when PhD delay leads to attrition/termination. Termination of the PhD trajectory can lead to individual stress, a loss of valuable time and resources invested in the candidate and can also mean a loss of competitive advantage. Using data from two studies of doctoral candidates in the Netherlands, we take a closer look at PhD duration and delay in doctoral completion. Specifically, we address the question: Is it possible to predict which PhD candidates will experience delays in the completion of their doctorate degree? If so, it might be possible to take steps to shorten or even prevent delay, thereby helping to enhance university competitiveness. Moreover, we discuss practical do's and don'ts for universities and graduate schools to minimize delays.

## Introduction

Universities across the globe are increasingly focused on how to be competitive in global and national rankings, and are often looking for ways to improve research and teaching efforts. The role of PhD candidates is extremely important in this regard as they can potentially produce a large amount of scientific output, a factor crucial in most ranking systems. The Shanghai Ranking, one of the most recognized academic ranking systems, ranks universities in part based on the number of successful PhD completions. A delay in PhD completion, while likely undesirable for PhD candidates, can also be detrimental to universities if PhD delay leads to attrition (i.e. termination of the PhD trajectory). PhD termination can lead to individual stress, a loss of valuable time and resources because of all the training and supervision invested in the candidate [1], and can also mean a loss of competitive advantage [2].

While many countries maintain a notional PhD duration of three or four years [3], in reality, PhD candidates often take much longer to complete their doctoral studies. Using data from two studies of doctoral candidates in the Netherlands, we take a closer look at PhD duration and delay in doctoral completion. Specifically, we address the question: Is it possible to predict which PhD candidates will experience delays in the completion of their doctoral degree? If this is possible, then it is also possible to take steps to shorten or even prevent delay, thereby helping to enhance university competitiveness.

### PhD completion in The Netherlands

The Dutch system of doctoral education has a number of characteristics specific to the Dutch context [4]. One important characteristic in relation to PhD completion and delay is the structure of funding and time given to PhD candidates to complete the PhD. Most PhD candidates are employed by the university for a set period of time to complete a PhD. The funding for these positions within the university often stems directly or indirectly from an external source, such as a research grant. As such, PhD projects consist primarily of a pre-specified trajectory of anywhere between three and five years. One consequence of this structure is that the contract duration for the PhD is set prior to a candidate starting a doctoral trajectory. Therefore, PhD candidates have no influence on the duration of the contract. Exceptions to this can only occur in special cases of delay, for example delay due to maternity leave or extended illness, or if a PhD candidate requests a decrease in working hours, which is a legislative right in the Netherlands. Individuals who have worked for their employer for 12 months or longer have a right to request an increase or decrease in working hours. If a business wishes to refuse such a request, the burden of proof is on employers to prove that granting the request would be harmful to the business. In these cases, the contract is likely to be extended pro rata to the time taken off work or the reduction in working hours.

It should be noted that the set time limit of the Dutch system does not mean PhD candidates cannot continue to work on the PhD thesis or graduate after the contract finishes. Rather, the set time limit refers to the period of time during which a candidate receives funding and can work (almost) full-time on the PhD thesis. Beyond this period, the candidate is responsible for finishing the thesis in his/her own time, which can lead to further delay. An advantage of this system is that PhD candidates have a period of guaranteed funding, during which they have the capacity to undertake field work, carry out research, and write, with minimal teaching obligations. While PhD candidates in the Netherlands may experience delays throughout the PhD trajectory, either within or beyond this set time period, these delays will most likely not be due to an absence of funding or the necessity of other professional work to finance one's PhD trajectory (for example teaching assistantships). This may not be the case with delays experienced by PhD candidates in other countries, such as the United States, where funding for doctoral research differs. What these different delays (financial, research-oriented, and supervisory) mean for PhD candidates and their success, and how this differs across countries, remains an important issue for further research.

Another characteristic of the Dutch system is that most PhD students are paid by way of the university as regular employees with a set salary level (set by collective agreement). While this is the case for most PhD candidates, it is not true for all of them. In the Netherlands, it is possible to differentiate between three different types of PhD status, including: (a) PhD candidates employed by the university (on the basis of university funding or external funding, such as funding from the national science foundation or third (private) parties), (b) scholarship recipients, and (c) external and/or dual PhD candidates. The first form is the exception and not the rule in most doctoral education programs in industrialized countries. The co-existence of multiple types of doctoral candidates is not unique to the Netherlands, however. Germany, Finland and Turkey also have doctoral systems where various types of PhD candidates co-exist, including PhD candidates employed by universities, scholarship recipients and external candidates who combine doctoral work with professional activities in other organizations [5]. What is unique about the Dutch system, however, is the high proportion of PhD candidates who are paid to work full-time or nearly full-time (0.8 FTE) on their research and PhD thesis. As noted above, a major advantage of this system is that by providing PhD candidates with a stable funding source, PhD candidates are often successful in completing the doctoral trajectory within the pre-set time period [6]. The average completion rate in the Netherlands, in general, is around 75 per cent. The existence of such a system is also useful for understanding PhD delay, a point we address below.

While the Dutch system provides most PhD candidates with a stable funding source, these external funding sources generally do not provide for the coverage of salary costs associated with an extension of a PhD contract. Therefore, any delay in the PhD trajectory in terms of salary costs has to be paid for by an academic department or institute. Alternatively, the PhD student must finish the thesis in his/her private time without drawing a salary from the university. Financially, delays can be costly for academic departments and are highly undesirable. If universities are not willing to cover the cost of an extension of the contract and a PhD candidate must finish the thesis in his/her own time, the risk increases that the thesis will not be completed [7]. In essence, the greater the duration of PhD delay, the greater the likelihood that a thesis may never be completed. Failure to complete the thesis translates into a significant loss in research investment and lost revenue for universities. In the Dutch case, this can also mean a significant financial loss because universities are rewarded financially by the government for PhD completions (€90,000 per successfully defended thesis).

### Predictors of PhD-delay

While most other studies investigating variation in PhD completion typically focus on describing causes of (high) attrition rates [8], predicting the timing of completion [2], [9], and/or time-to-degree [10], [11] given the structure of the Dutch system we are able to measure the ‘true’ rate of delay. Rather than merely attempting to predict the timing and duration of PhD completion and/or time-to-degree, the structure of the Dutch system means we know *a priori* how long a PhD should take (expected duration) versus the actual duration. The expected duration is equal to the pre-determined end date minus the pre-determined starting date, whereas the actual duration is equal to the actual end date minus the actual starting date. The difference between these two is what we call ‘delay’. This measurement of the true rate of delay means we can focus on which factors predict PhD delay. Explanations for variation in PhD completion rates and/or time-to-degree can be sought in a number of areas and are often difficult to disentangle, but can be generalized into three categories [6], [8], [9], [11]–[16]:

1. *Institutional or environmental factors*, including field of study, departmental research climate, and resources and facilities available to the project;
2. *The nature and quality of supervision*, entailing both the frequency of meetings as well as the support of research colleagues;
3. *Characteristics of the PhD candidate*: including gender, ethnicity, age, having children, marital status, satisfaction with the project, academic achievement, and expectations about the project. In addition, certain personality traits, such as patience, a willingness to work hard, motivation and self-confidence have also been shown to influence PhD completion rates, but accounting for variation in these traits is beyond the scope of the research design here.

Factors most important in determining delay vary across university settings but some key warning signs, as noted by [17], are:

- constant changes to the research topic;
- avoiding communication with the supervisor;
- PhD candidates isolating themselves;
- avoiding submitting work for review.

The above findings have, to our knowledge, never been included in a single quantitative study, which we ascribe to do here.

### Gender

Before discussing the data and methodology, we call attention to one possible factor of interest: gender. Recent educational statistics show that women are increasingly taking part in higher education, including doctoral education [18]. Whereas a previous study conducted in the Netherlands in 1995 found that one fifth of PhD candidates were female [19], a more recent study conducted in 2008–09 shows that this percentage has more than doubled to 47 per cent [20]. The effect of gender on the duration of the PhD trajectory is, however, disputed. While some studies find gender differences [21], others do not [15], [22]. Some studies report a positive relationship between being married or having children and delays in PhD completion for women [8], however others suggest the effects of being married and having children are usually larger for men, as the behavioural changes accompanying marriage and parenthood are smaller for women than for men [23]. A recent article in *Nature* confirms the contradictions evident in research that investigates gender differences in relation to the PhD trajectory [24]. We address this issue by predicting PhD delay separately for male and female PhD candidates.

### Data

In the current paper we use data from two separate but related studies. While these studies are drawn from different populations and use various methods, they allow for a closer examination of PhD duration and delay in the Netherlands. We discuss the generalizability and possible limitations of these studies in our conclusions. The first dataset stems from a survey of doctoral recipients who completed their PhD in 2008–2009. Using these data we a) describe the occurrence of PhD delay and b) build a statistical model to predict which PhD candidates are likely to be delayed. The second dataset consists of PhD candidates surveyed in The Netherlands at Utrecht University in the final year of their PhD. These candidates were asked whether they expected to complete their PhD on time. Candidates expecting to be delayed were asked about possible reasons for this delay, including a number of open-ended questions. Data from this study allow us an opportunity to contextualize delays in PhD completion experienced by doctoral candidates. We provide a further discussion of the data and methodology for each study and turn to the results of each of these studies below.

It should be noted that the research discussed here has not been subjected to an ethics approval process. While obtaining ethics approval is standard practice in most Anglo-American systems, this is not (yet) the case for most social science research in the Netherlands. In our case, no approval by an ethical review committee was obtained because the planned surveys with adult academics are neither physically nor emotionally burdensome nor do they violate respondents' privacy. We did obtain consent from each of the local executive boards at participating universities, however, and the research was undertaken with the utmost care. This includes, but is not limited to, ensuring the privacy and confidentiality of respondents, explaining the research process to participants and minimizing the demands placed on respondents by using well-tested survey instruments. Research was not undertaken outside the country of residence, therefore no local authorities were contacted. The research was not conducted in relation to any medical facility. The quantitative and qualitative data presented here are not publicly available. However, a copy of the fully-anonymized quantitative dataset is available from the first author upon request.

## Methods Study 1: PhD Duration and Completion

### Participants

The first study relies on survey data on Dutch doctoral recipients gathered between February 2008 and June 2009 (response rate 50.7%; n = 565; 47% female; 73.8% were of Dutch origin) in the Netherlands at four universities (Delft University of Technology, Erasmus University Rotterdam, Utrecht University, and Wageningen University and Research Centre). For more details see [25].

Of the 565 respondents surveyed, the majority (71.1%) reported that their formal PhD status was ‘employee’ at a university with five per cent listing ‘scholarship recipient’ as their main PhD status. The share of external or dual PhD candidates was 23.9 per cent. In the current paper we focus solely on those respondents who reported their start and end date, and who reported their status as being an employee (n = 308) or scholarship recipient (n = 25), of which 48 per cent were female. This decision is based on the transparency of these PhD trajectories. PhD candidates employed by the university and scholarship recipients have unambiguous start and end dates and these candidates primarily work full-time on their PhD thesis, allowing for a clear look at PhD delay. The group of PhD candidates not employed by the university is highly heterogeneous, which makes it difficult to assess delay clearly. There were no significant differences on key background variables between respondents included/excluded from our study. The total sample size used for the analyses is therefore n = 301 and a summary of descriptive statistics on this sample can be found in Table 1. Note that we also deleted two outliers because they reported unrealistic values for the gap between actual and completed project time, namely -31 (completed the PhD 31 months sooner than expected) and 91 (completed the PhD 91 months later than expected). We conducted the final analyses with and without these two cases and although some numerical differences appeared, our conclusions remained the same.

**Table 1:** Sample Characteristics Study 1.

	Females^a^				Males^b^			
Variable		*%*				*%*		
Dutch passport (%)		67.7				67.1		
Change in marital status during PhD trajectory (%)		31.6				27.7		
Marital status at end of PhD trajectory (%)								
Never married/divorced/widowed/separated		33.8				36.4		
Children under 18 living in the household (%)		13.3				22.5		
Change of supervisor, institute, or thesis topic (%)		28.5				23.1		
Conference attendance (%)		83.5				82.1		
		95% CI				95% CI		

Variable	*M* (SD)	*LL*	*UL*	*M* (SD)	*LL*	*UL*	
Age	31.07 (6.03)	29.99	32.16	31.05 (6.26)	29.94	32.16	
Number of articles submitted for publication	5.15 (3.58)	4.50	5.79	4.78 (3.57)	4.15	5.42	
Number of articles accepted for publication	4.01 (3.04)	3.46	4.56	4.12 (3.01)	3.59	4.65	
Number of supervisor expectations in publishing	3.25 (1.71)	2.94	3.56	3.14 (1.60)	2.86	3.43	
Supervisor(s) provided good opportunities for establishing international contacts	2.20 (1.08)	2.00	2.39	2.18 (1.07)	2.00	2.37	
Having a clear idea of data needed to answer research questions prior to start of the second year of the PhD trajectory	2.92 (1.25)	2.69	3.14	2.69 (1.17)	2.48	2.89	
Taking part in numerous group projects during the PhD trajectory	2.93 (1.03)	2.75	3.12	2.73 (1.19)	2.52	2.94	
Gained extra research experience during the PhD trajectory, including experience on research projects outside of own thesis topic	2.74 (1.19)	2.53	2.96	2.33 (1.11)	2.13	2.52	
Supervisor(s) encouraged me to publish in international scientific journals during the PhD trajectory	1.41 (0.78)	1.27	1.55	1.57 (0.87)	1.41	1.72	
Within my PhD research, it was necessary to work with other PhD candidates, both within and outside graduate or research schools	2.53 (1.14)	2.32	2.73	2.60 (1.24)	2.38	2.82	
Supervisor(s) felt it was important to finish the thesis in a timely manner, particularly in relation to job prospects following graduation	2.74 (1.15)	2.54	2.93	2.08 (1.09)	2.61	2.99	
Visited conferences with supervisor(s), which improved contacts with potential employers	2.84 (1.25)	2.62	3.07	2.65 (1.21)	2.43	2.86	
Having a clear idea of the theoretical and/or societal relevance of research topic from the start of the PhD trajectory	2.58 (1.08)	2.38	2.77	2.22 (0.92)	2.05	2.38	
Knowing precisely which research questions the candidate wants to answer at the end of the first year of the PhD trajectory	3.17 (1.19)	2.95	3.38	3.00 (1.10)	2.81	3.19	
Succeeding in determining methods of data collection needed to gather data after clarifying research questions	2.13 (0.86)	1.98	2.29	2.02 (0.76)	1.89	2.16	
Supervisor/s gave good advice on topic selection and refinement	2.12 (1.00)	1.94	2.30	2.21 (0.97)	2.04	2.38	
Receiving excellent guidance in the search for relevant literature	2.69 (1.06)	2.50	2.88	2.67 (1.11)	2.48	2.87	
Considering maintaining professional contact with a number of former PhD colleagues as highly likely	1.93 (0.84)	1.78	2.09	1.87 (0.84)	1.72	2.02	
Supervisor(s) emphasized independence	1.95 (0.85)	1.80	2.10	1.86 (0.84)	1.71	2.00	

*Note*. CI  =  confidence interval for mean; *LL*  =  lower limit, *UL*  =  upper limit.

a *n* = 121. ^b^ *n* = 12.

### Procedure

All PhD candidates who applied for permission to defend their thesis were invited to participate in the survey. Respondents were contacted through the Registrar's office (the *pedel)*, the university office in charge of organising the doctoral defence, at each of the participating universities. Note that in the Netherlands the so-called ‘all-but-dissertation’ (ABD) status does not exist, and registering for the defence is only allowed after official approval of the doctoral thesis by the defence (examination) committee. Outside of exceptional cases such as fraud, the degree will be conferred following a primarily ceremonial defence. When PhD candidates registered for their defence, they received an informational packet, which included a letter from the university Board of Governors (*College van Bestuur*) explaining the aim and objectives of this research project and asking for their participation. The Netherlands Centre for Graduate and Research Schools was then provided with a list of e-mail addresses of PhD candidates who registered for the defence at each university. Respondents were approached within 10–14 days after registering for graduation and were provided a login and password to complete the survey. Up to two reminder emails were sent if a respondent did not sign in to complete the survey. In sum, respondents received a maximum of three e-mails asking them to participate. Any identifying information has been removed from the data for purposes of confidentiality.

### Measures

We asked the participants to provide information on certain background characteristics such as age, gender, citizenship (whether or not they were born in the Netherlands and/or have a Dutch passport), marital status (including cohabitation), both as a static category and whether their marital status changed during the PhD trajectory, and whether there are any children under the age of 18 living in the household. Furthermore, we asked them questions about any major changes occurring during the PhD trajectory. These changes included: [Did you change']… ‘[…] your main supervisor?’ '[…] daily supervisor?’ ‘[…] the institute or graduate school where you were completing the PhD?’, and ‘Did you change your thesis topic?’ In addition, respondents were asked about their publication record, including the number of submitted and accepted articles as well as conference visits. We then asked about perceived expectations from supervisors, including the expected number of journal articles, book chapters, conference papers, conference visits, etc. Finally, we asked respondents to reply to 15 statements about their supervisor and the academic climate in their department. Answers were scored on a 5-point Likert scale ranging from strongly disagree to strongly agree. One example of these statements is ‘Prior to the start of the second year of my PhD trajectory, I had a clear idea which data I would need to answer my research questions’. All 15 statements can be found in Table 1. Each of these predicting variables was added to the model in one step. In addition, we control for the relationship between age and having children by regression the variable having children on age, see also the syntax in the supplementary materials.

### Statistical Analysis

As discussed above, the Dutch system is characterized by having PhD trajectories with primarily fixed durations. Consequently, a PhD project includes a pre-determined start and end date which makes it possible to compute an exact duration for the PhD, both actual and expected. In the survey, all respondents were asked to indicate the length of their contract (planned PhD duration) as well as how long it took them to complete their thesis (actual PhD duration). This information can then be used to compute the average gap between actual and planned duration, referred to as the *gap*.

Using the *gap* as our dependent variable, we can build a statistical model where we add predictors of the average *gap* for females and males separately. We provide the syntax of the model in the appendix (see Appendix S1), and the data can be requested by sending an email to the first author. We have used Bayesian statistics in the software package Mplus v7.0 [26], [27] for all of the analyses. Mplus is a software package that can deal with many types of statistical models with continuous and categorical variables and different types of estimators, for example maximum likelihood, weighted least squares, bootstrapping and the Bayesian estimator. Bayesian statistics are becoming more common in academic research [28]. The number of papers published, for example, in the journal *PLoS One* with Bayes in the title or abstract has increased from only one in 2006 to 89 in 2011. The key difference between Bayesian statistics and ML-estimation concerns the nature of the unknown parameters. For example, following the frequentist framework approach to maximum likelihood estimation, a parameter of interest is assumed to be unknown, but fixed. That is, it is assumed that there is only one true population parameter in the population; for example, one true regression coefficient. In the Bayesian view of subjective probability, all unknown parameters are treated as uncertain and should therefore be described using a probability distribution. Hence, with Bayesian statistics, all parameters of the model (e.g., means, variances, regression parameters, etc.) are repeatedly estimated in an iterative process. This distribution of parameters can subsequently be used to compute the mean regression coefficient and its confidence interval. For a more detailed comparison and for an introduction to Bayesian statistics see the many textbooks on this topic, for example [29].

**Figure 1:**
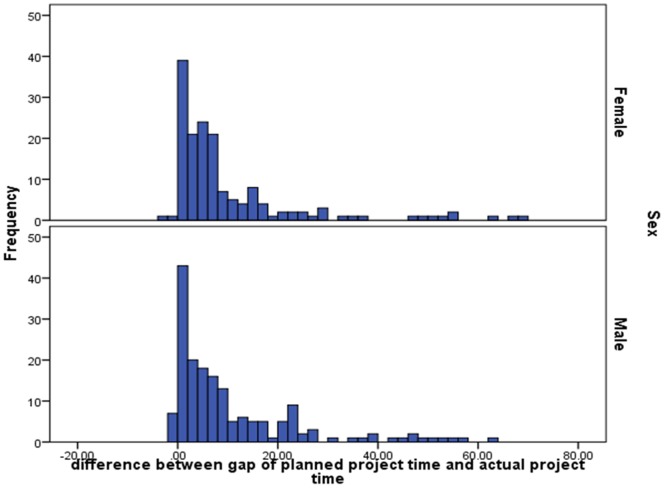
The difference between planned and actual PhD duration in months for male and female PhD candidates (separate figures, by gender).

In our case there are three main reasons why we have chosen to use Bayesian statistics. First, Bayesian estimation is less sensitive to the distribution of the parameters in our model because of the iterative process. This is an advantage in our case because of the highly skewed distribution of our dependent variable (see Figure 1). Second, in each iteration of the iterative process, missing data is automatically imputed. In our data, 75 per cent of the cases had complete data and another 20 per cent had missing data for only one or two variables. The remaining 5 per cent had missing data on multiple variables. The amount of missing data was not related to any of the variables in the model. Third, the use of Bayesian statistics results in slightly different interpretations of the results compared to maximum likelihood or a weighted least squares estimation. When Bayesian statistics are used, the confidence intervals (i.e., credibility intervals, or posterior probability intervals) are used to indicate the 95 per cent probability that the estimate will lie between the lower and upper value of the interval. When the interval does not include zero, the null hypothesis is rejected and the effect is assumed to be present.

On a final methodological note, when analyzing statistical models, we may be interested in more than just confirming or rejecting a single hypothesis –we may want to evaluate the entire model. When using Bayesian statistics, classical model fit indices, such as the CFI, TLI, and RMSEA are not available. However, it is possible to obtain the predictive accuracy of the model (see [30] for a more detailed discussion). This evaluation of the model is also referred to as posterior predictive checking, see [31]. In Mplus, the posterior predictive *p*-value (*ppp-value*) is given and *ppp*-values around.50 indicate a good-fitting model.

## Results Study 1: PhD Duration and Completion

**Figure 2:**
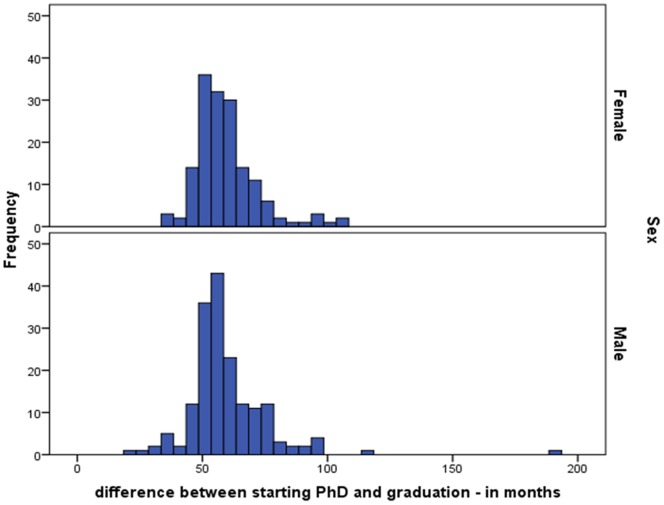
Time difference between starting the PhD and thesis defence in months for male and female PhD candidates (separate figures, by gender).

Starting with results from our first study, the data show that female PhD recipients took an average of 59.8 months (95% CI: 57.18–61.82) to complete their PhD thesis and male PhD candidates an average of 59.67 months (95% CI: 57.46–61.91), see also Figure 2. The average gap between actual and planned duration (i.e., the *gap*) was 9.52 months for women (95% CI: 7.43–11.69) and 10.11 months for men (95% CI: 8.09–12.17), see also Figure 1. Since the 95% CI for both variables for women and men completely overlap, no significant gender differences are found. While the duration of the *gap* does not differ for men and women, we do find significant differences in what causes the *gap*, or rather what is associated with the *gap*. Because our data is cross-sectional data, we cannot make assumptions about causal relationships.

In the statistical model for female PhD candidates (n = 158), 30.0 per cent of the variance in the *gap* was explained and the *ppp-value* is.60, indicating a well-fitting model. Our results clearly show significant predictors, that is, the 95 per cent CI does not include zero, see Table 2. For women, a change in marital status during the PhD trajectory (while controlling for the status itself) is associated with more than five months delay. In addition, having had opportunities through their supervisors to establish international contacts was associated with a three month delay. In contrast, for women, working together with other PhD candidates is associated with a four month gain in project time.

**Table 2:** Bayesian Results for the Multiple Group Model Explaining the Gap between Actual and Planned PhD Duration.

	Model female PhD candidates^a^			Model male PhD candidates^b^		
Variable	*B* (SD)	95% C.C.I.		*B* (SD)	95% C.C.I.	
Age	0.23 (0.23)	−0.21	0.68	−0.09 (0.16)	−0.40	0.22
Dutch passport	1.39 (2.79)	−4.12	6.29	4.39 (2.39)	−0.32	9.04
Change in marital status during PhD trajectory	**5.40 (2.67)**	**0.17**	**10.60**	−1.60 (2.90)	−7.30	4.08
Marital status at the end of the PhD trajectory	2.63 (2.81)	−2.95	8.13	0.18 (2.75)	−5.24	5.65
Children under 18 living in the household	−0.53 (1.87)	−4.28	3.04	**3.71 (1.47)**	**0.79**	**6.56**
Children under 18 living in the household ON age	**0.06 (0.02)**	**0.02**	**0.09**	**0.03 (0.01)**	**0.01**	**0.06**
Change of supervisor, institute, or thesis topic	−0.78 (2.60)	−5.81	4.35	**5.60 (2.67)**	**0.40**	**0.88**
Number of articles submitted for publication	−0.70 (0.48)	−1.63	0.24	0.08 (0.44)	−0.79	0.96
Number of articles accepted for publication	−0.01 (0.58)	−1.13	1.14	−0.27 (0.54)	−1.34	0.79
Conference attendance	−2.29 (3.32)	−8.80	4.27	−**7.18 (2.92)**	−**12.92**	−**1.46**
Number of supervisor expectations	−0.19 (0.74)	−1.64	1.27	−0.32 (0.71)	1.72	1.09
Supervisor(s) provided good opportunities for establishing international contacts	**3.34 (1.33)**	**0.73**	**5.93**	1.30 (1.37)	−1.42	3.99
Having a clear idea of data needed to answer research questions prior to start second year of PhD trajectory	−0.12 (1.31)	−2.68	2.47	2.58 (1.55)	−0.48	5.64
Taking part in numerous group projects during PhD trajectory	0.69 (1.24)	−1.73	3.13	1.13 (1.14)	−1.10	3.36
Gained extra research experience during PhD trajectory, including experience on research projects outside of own thesis topic	−0.61 (1.05)	−2.66	1.45	−0.45 (1.18)	−2.76	1.89
Supervisor(s) encouraged me to publish in international scientific journals during PhD trajectory	−1.15 (1.54)	−4.16	1.90	−0.11 (1.38)	−2.81	2.61
Within the PhD research, it was necessary to work with other PhD candidates, both within and outside graduate- or research school	−**4.34 (1.19)**	−**6.68**	−**2.02**	−1.00 (1.08)	−3.13	1.09
Supervisor(s) felt it was important to finish the thesis in a timely manner, particularly in relation to job prospects following graduation	0.91 (1.26)	−1.59	3.36	−1.08 (1.10)	−3.20	1.11
Visited conferences with supervisor(s), which improved contacts with potential employers	−1.56 (1.26)	−4.05	0.90	−1.07 (1.10)	−3.19	1.09
Having a clear idea of the theoretical and/or societal relevance of the research topic from the start of PhD trajectory	1.53 (1.31)	−1.03	4.09	−0.35 (1.32)	−2.92	25
Knowing precisely which research questions the candidate wants to answer at the end of first year of PhD trajectory	−0.61 (1.41)	−3.36	2.17	−**3.85 (1.56)**	−**6.91**	−**0.79**
Succeeding in determining methods of data collection needed to gather data after clarifying research questions	1.44 (1.86)	−2.23	5.05	−1.91 (1.64)	-5.13	1.29
Supervisor/s gave good advice on topic selection and refinement	−1.27 (1.82)	−4.80	2.34	2.79 (1.80)	−0.80	6.28
Receiving excellent guidance in the search for relevant literature	−0.66 (1.62)	−4.05	1.99	−2.20 (1.43)	−4.96	0.62
Considering maintaining professional contact with a number of former PhD colleagues as highly likely	−0.57 (1.51)	−3.57	2.37	−0.29 (1.59)	−3.40	2.82
Supervisor(s) emphasized independence	0.87 (1.48)	−1.99	3.78	1.26 (1.41)	−1.49	4.08

*Note*. Central credibility intervals (95% C.C.I.) that do not include zero are presented in bold.

a *n* = 158. ^b^ *n* = 173.

In the statistical model for male PhD candidates (n = 173), 30.4 per cent of the variance in the *gap* was explained and the *ppp-value* is.52, also indicating a good-fitting model. In contrast to women, marital status was not associated with the *gap* for men, but having children is associated with almost four months delay. Moreover, for men, a change of supervisor or thesis topic was associated with a five-and-a-half month delay. Conference attendance, however, was associated with a decrease of the *gap* by 7 months. In addition, for men, whether the PhD candidate knew which research question to answer by the end of the first year was associated with a 3.8 month decrease in the gap.

## Methods Study 2: Explaining PhD Delay

### Participants

The second study relies on survey data on doctoral recipients gathered in 2010 at Utrecht University in the Netherlands, for more information see [32]. The sampling frame included all PhD candidates registered at Utrecht University. In other words, the frame consists of candidates employed by the university as well as external and dual PhD candidates (candidates who combined a PhD with another job or other activities), and scholarship-funded PhD candidates. Candidates were invited to rate various aspects of their PhD experience through an online questionnaire, including a series of open ended questions. In total, 2,870 candidates were approached and of these 2870 candidates, 1,504 (52%) completed at least one part of the survey. Similar to the previous study, most PhD candidates surveyed (79%) were employed by the university, 5% of respondents were on a PhD scholarship and external and/or dual PhD candidates (who combine a PhD with other activities) made up 12 per cent of the candidates surveyed. Nearly one third (31%) of the respondents had a non-Dutch nationality. The top three foreign nationalities included German (3%), Italian (3%) and Chinese (2%). Candidates' average age was 31. More than one–third of candidates (36%) were older than 31. Fifty-seven percent of the candidates were female and 43 percent were male.

In line with the previous study, while a survey carried out at one university in the Netherlands may not be representative of the population of PhD candidates as a whole, the data provide rich, contextual data on expectations of PhD duration and reasons for delay.

## Results Study 2: Explaining PhD Delay

Using data from this second study, it was possible to determine the current stage of the PhD trajectory for 1,286 respondents: 25 per cent were in their first year, 19 per cent were in their last year, 53 per cent were somewhere in between and 3 per cent had recently graduated. When asked whether they were on track to finish their PhD thesis on time, 60.5 per cent of the PhD candidates reported they expected to finish on time, while 27.5 per cent expected difficulty in finishing on time and another 12 per cent did not know. If we select only those PhD candidates who were in the final year of their PhD, 88 out of 232 (38%) expected to experience problems in finishing on time. For the remainder of the analysis, we refer only to this group of respondents in the final year of their PhD. Not only do these candidates probably know best *why* they were experiencing a delay (the time to finish their PhD was quickly running out), it is also plausible that an expected delay in the first few years of the PhD trajectory may be resolved at a later stage. Due to the small sample size, we do not exclude external and/or dual PhD students from this study, whereas external and/or dual candidates are excluded from Study 1.

**Figure 3:**
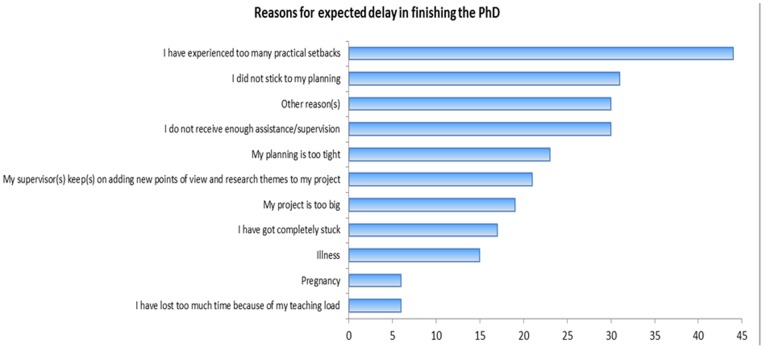
Possible reasons for a delay in finishing the PhD.

Respondents were asked about the reasons for the expected delay and could choose from ten answer categories, see Figure 3. Multiple answers could be provided. Responses to this question illustrate that experiencing practical setbacks is the most common reason for a delay, followed by not adhering to the original thesis plan. In contrast to other countries like the US, Dutch PhD candidates do not wait to select a thesis topic until later in the PhD trajectory. Rather, PhD candidates start their trajectory with a clear topic and research plan laid out.

We also asked respondents a number of open-ended questions about expected delays. The responses to these questions can be grouped into four broad themes thought to influence delay:

- *Thesis-related issues*, meaning additional work needed to be done (*n* = 16), such as extra papers being written or statistical analyses taking longer than expected; bad planning or a change in plans, and external circumstances (*n* = 15) such as waiting for donor material, waiting for ethics approval, or as one respondent replied, “experiments were affected due to renovations in the building”.
- *Supervisor-related issues*. For many respondents, clear guidance and communication were essential to their PhD trajectory (*n* = 17). Stated differently, an absence of clear guidance and communication were seen as integral in explaining their expected delay.
- *Personal issues*. This includes circumstances at home *(n* = 15), such as care responsibilities, or more serious circumstances such as the death of a relative, a candidate suffering from severe illness; or personal difficulties in managing the project (*n* = 8).
- *Combination problems*. These issues involved trying to combine the PhD with other duties, such as other work (n  = 24); starting a new job before finishing the thesis; or as one respondent replied, needing “to spend time pleasing the grant provider.”

For many respondents, clear guidance and communication were essential to their PhD trajectory. Or rather, they perceived an absence of clear guidance and communication as fundamental in causing delay, as these two respondents discuss:

*“I have been having a difficult time relating with my first project which I started with my supervisor, who moved to another institute and who doesn't pay attention to what I am doing anymore. [...] I fell in a void when my previous supervisor left, and no one noticed. It took me 1.5 years to find a new supervisor, start a project etc. That time is lost, and I do not get any (monetary) help on that point.”*

*4^th^ year PhD candidate in the Social Sciences, delayed by 6 months and still working on the thesis*

*“My supervisor does not motivate or stimulate me scientifically or socially. He does not provide any practical supervision, nor does he ensure that a secondary supervisor does so, even when explicitly asked to do so and agreeing upon this. This has caused considerable and unnecessary delay in my project. When confronted, the supervisor denies any insufficiencies and does not show willingness to invest in improving the situation.”*

*4^th^ year PhD candidate in the Health Sciences delayed by approximately 1 year*

The frustration caused by an absence of clear guidance and communication is summed up succinctly by the response of one PhD candidate who stated:

*“HE'S LEFT ME ALONE”. (Emphasis in original)*

*4^th^ year PhD candidate in the Earth Sciences delayed by approximately 2 years*

Answers to these open-ended questions provide interesting insights into PhD candidates' experiences and perceptions of delay. Together with the results from the first study, the data offer a starting point for developing practical tips for preventing delay. One creative and useful way of developing these tips is to apply the Machine Trick to these responses, suggested by famed sociologist Howard Becker [33]:

*Take a second. Imagine that you have a spouse/partner. We ask you to tell us what your partner should do to keep you happy. You could talk for hours, mentioning dozens and dozens of examples of what the partner should or should not do. Now we apply the Machine Trick. What should your partner do to make you feel sad and unhappy as quickly as possible? Within five minutes you will be able to sum up the essential things, the opposite of which thus provides key insights into having and maintaining a happy relationship.*

To understand key factors contributing to the successful completion of a PhD project, we should ask ourselves what key factors a “machine” would use to make a PhD project fail. Of course, as Becker tells us, in actuality we do not want a PhD project to fail. But utilizing such a machine-designing exercise offers a systematic means of considering which factors contribute to the failure (and conversely the success) of a PhD project.

Applying Becker's Machine Trick to our qualitative data, we can conclude that key steps likely to contribute to the failure of a PhD project include:

- Admit doctoral candidates who demonstrate the least amount of knowledge about their potential PhD topic.
- Base admission decisions on written material only – do not invite candidates for face-to-face interviews.
- Do not test the (English) language proficiency of PhD candidates from abroad.
- Restrict supervision to one supervisor who is overloaded with responsibilities, has multiple PhD candidates and offers no team supervision.
- Restrict supervision to a supervisor who does not care about PhD planning, who will meet with the candidate once every two or three months at the most and who will let the PhD candidate independently determine which criteria are applied in assessing the thesis and if/when progress will be monitored.
- Do not assess whether the candidate possesses the basic and necessary qualities for designing and completing a PhD project prior to enrolment.
- Have the candidate focus solely on reading and do not provide any training in rigorous, academic writing or any other research skills.
- Isolate the candidate: Communication with other experts or peers to discuss one's work should be avoided.
- And please, let the candidate teach for at least for three or four days a week.

These factors will guarantee a delay of the PhD candidate. While these tips may appear self-evident, few studies offer empirical evidence from the perspective of PhD candidates on which to base these recommendations. While further research is needed to test the generalizability of the results shown here, taking steps to develop policies aimed at addressing these concerns can minimize the chances of delay.

## Discussion

In this paper, we have taken a brief look at PhD delay. Results from the first study show significant gender differences in predicting PhD delay, confirming findings from [21]. *What* is associated with delay differs for men and women. For women, work and social contacts are associated with a reduction in delay, whereas for men, conference attendance and knowing precisely which research questions the candidate wants to answer at the end of the first year is associated with a decrease in delay. We also find that for women, a change in marital status (while controlling for marital status itself), and having had opportunities through their supervisors to establish international contacts are associated with delay. For men, having children younger than 18 in the household or experiencing a change of supervisor or thesis topic is associated with a delay in finishing the PhD. In part, then, our results appear to confirm findings from Waite [23], that the effects of having children are larger for men than for women. In fact, we find no significant effect of having children under the age of 18 on the PhD delay experienced by women. The absence of a finding here could be a reflection of when women choose to have children. Mastekaasa [34] finds, for example, that there is no relationship between having children and completion rates of doctoral candidates in Norway, as long as children were born prior to commencement of a PhD program. Female doctoral candidates may make a conscious choice to delay childbearing until after PhD completion. However, more research is needed to determine the validity of such an argument.

The second study, looking in more detail at reasons for expected delays, demonstrates that practical setbacks can lead to unnecessary delays in the PhD trajectory. This may not be a surprising finding, given that practical setbacks, such as problems with data, are part of doing research more generally and the PhD experience in particular. However, an individual's ability to deal with these practical setbacks may be what separates a successful scientist from a less successful one. In addition, the open-ended responses provided by PhD candidates in the second study suggest that universities and graduate schools can work with PhD candidates to minimize these delays by:

- ensuring PhD planning takes place within a reasonable timeframe;
- by conducting structural reviews of PhD progress;
- working to ensure effective communication between candidates and supervisors;
- and providing structural support to PhD candidates, for example support for those individuals with caring duties.

We note a number of limitations, however. Our studies were conducted in the Netherlands, and while the Dutch system provides a clear-cut case for examining PhD delay, the PhD system in the Netherlands may not necessarily share characteristics common to doctoral systems in other countries. Internationally comparable data would be welcome in this regard. In addition, we have not been able to control for the diversity in funding sources. The source of funding for a PhD project may be directly or indirectly related to experienced delays. For example, PhD supervisors may be involved as Principle/Chief Investigators on multiple projects, which can lead to reduced time for PhD advising and supervision, which can lead to delay. Conversely, certain funding sources may require regular updates and have structures in place which help to prevent delay. Future research that can account for variation in funding is needed to investigate this further.

Despite these limitations, the results presented here offer important insights for universities and graduate schools. A major lesson we can take from this research is to evaluate the work of potential PhD students before they start their PhD trajectory. The necessity for such an evaluation is one reason that many European graduate schools are considering or have already implemented special tracks within Master degree programs that allow for the development and evaluation of potential PhD research proposals prior to any undertaking of a PhD trajectory. This often occurs in cooperation with a potential supervisor. In this manner, the qualities of the potential candidate can be evaluated before either the candidate or the graduate school invests further time and money into a (sometimes lengthy) PhD trajectory. It can also be a means of testing the working relationship between a candidate and their potential supervisor. An essential component of this approach, however, is that students participating in these special tracks still compete for a position as a PhD candidate. While a proposal developed during a special Master track might be of high quality, this quality should be tested in relation to other candidates applying for the same position.

But more importantly, our results indicate that it is possible to predict which PhD candidates will be delayed:

- Female PhD candidates who experience a change in marital status;
- Female PhD candidates who invest time in international contacts;
- Male candidates with children;
- Male candidates who experience a change in supervisor;
- Candidates who experience practical setbacks (such as problems with data collection);
- Candidates who do not adhere to the original thesis plan;
- Candidates suffering from the absence of clear communication with and guidance from their supervisor(s); and
- Candidates with extenuating personal circumstances.

Of course these findings have to be replicated over time, across countries and in different university settings, but they provide a starting point for policy recommendations. The delays in PhD projects are not inevitable; universities and graduate schools would be well placed to investigate further the reasons for delay and steps that could be taken to minimize this delay. Taking steps to avoid the “machine-generated” fail factors can improve PhD completion rates and reduce PhD delay. Such improvements are not only beneficial to individual PhD candidates, but on a more aggregate scale can lead to an improvement in university competitive advantage and global rankings.

## Supporting Information

Appendix S1 **Mplus Syntax.**
(DOCX)
