# Supplementary material for: What Took Them So Long? Explaining PhD Delays among Doctoral Candidates
Source: PLoS One. 2013 Jul 23;8(7):e68839. doi: 10.1371/journal.pone.0068839 (PMC3720867; doi:10.1371/journal.pone.0068839)
Supplement: Appendix S1 — Mplus Syntax. (DOCX) [file pone.0068839.s001.docx]

APPENDIX S1: Mplus Syntax

DATA: FILE IS defdata.dat;

DEFINE: center E22_Age (grandmean);

VARIABLE:

NAMES ARE ID B3_GAP E4_having_child E22_Age B13_change_yes_no B20_Submitted_articles B20_Accepted_articles B23_conference_attendance expect_supervisor_count E2_maritalstatus_changed E2_maritalstatus_after_category E18_country_birth item1-item15;

USEOBSERVATION ARE (E21_sex EQ 0 AND outlier EQ 0); !0 = female 1 = male

MISSING are all (-999);

categorical is E4_having_child ;

USEVARIABLE are B3_GAP E4_having_child E22_Age B13_change_yes_no B20_Submitted_articles B20_Accepted_articles B23_conference_attendance expect_supervisor_count E2_maritalstatus_changed E2_maritalstatus_after_category E18_country_birth item1-item15;

ANALYSIS:

estimator = bayes;

bconvergence = .01;

chain = 10;

biterations = 500000(5000);

processor = 10;

MODEL:

B3_GAP ON E4_having_child E22_Age B13_change_yes_no B20_Submitted_articles B20_Accepted_articles

B23_conference_attendance expect_supervisor_count E2_maritalstatus_changed E2_maritalstatus_after_category

E18_country_birth item1-item15;

!to control for the relation between having children and age

E4_having_child on E22_Age;

!to estimate all correlations between x-variables

E4_having_child E22_Age B13_change_yes_no B20_Submitted_articles B20_Accepted_articles

B23_conference_attendance expect_supervisor_count E2_maritalstatus_changed E2_maritalstatus_after_category

E18_country_birth item1-item15;

OUTPUT:

stand tech1 cinterval;

PLOT:

type is plot2;
